# Recent Advances in Presentation, Diagnosis and Treatment for Mixed Vaginitis

**DOI:** 10.3389/fcimb.2021.759795

**Published:** 2021-11-02

**Authors:** Wenhui Qi, Huanrong Li, Chen Wang, Huiyang Li, Bingbing Zhang, Mengting Dong, Aiping Fan, Cha Han, Fengxia Xue

**Affiliations:** ^1^ Department of Gynecology and Obstetrics, Tianjin Medical University General Hospital, Tianjin, China; ^2^ Tianjin Key Laboratory of Female Reproductive Health and Eugenic, Department of Gynecology and Obstetrics, Tianjin Medical University General Hospital, Tianjin, China

**Keywords:** aerobic vaginitis, desquamative inflammatory vaginitis, cytolytic vaginosis, mixed vaginitis, vulvovaginal candidiasis, bacterial vaginosis, trichomoniasis, mixed biofilms

## Abstract

Mixed vaginitis is the simultaneous presence of at least two types of vaginitis, contributing to an abnormal vaginal milieu and leading to vaginal symptoms and signs. However, associations between symptoms and the type of mixed vaginitis have not been clearly elucidated, and research on mixed vaginitis is still in the preliminary stage. Therefore, the pathogenic mechanism of mixed vaginitis remains understudied. Mixed vaginitis generally involves the formation of mixed biofilms. The study of polymicrobial interactions and mixed biofilms will provide a new idea for the understanding of mixed vaginitis. Moreover, this review summarizes some effective management and laboratory diagnosis of mixed vaginitis to avoid inappropriate therapy, recurrence, and reinfection. It is of high clinical importance to obtain relevant clinical data to improve clinical knowledge about mixed vaginitis.

## Introduction

Mixed vaginitis is the simultaneous presence of at least two types of vaginitis, contributing to an abnormal vaginal milieu and leading to vaginal symptoms and signs. Nevertheless, individual signs and symptoms have only limited value in the recognition of vaginitis in clinical practice. For example, in patients with simple vaginitis, “vulvar pruritis” and “thick curdy discharge” are more likely to be reported by women with vulvovaginal candidiasis (VVC), while “thin white discharge” and “odor” are more commonly reported by women with bacterial vaginitis (BV) (Rivers et al., 2011). However, “abnormal vaginal discharge,” “dyspareunia,” and “vaginal soreness” can occur with any kind of vaginitis. The presentation of mixed vaginitis can be atypical. Consequently, simply identifying the presence of at least two types of vaginitis does not establish a cause–effect relationship with clinical symptoms and signs. The concept of mixed vaginitis has escaped clinical scrutiny and definition.

Today, approximately 20 lower genital tract-related infections have been recognized, and such infections are caused by bacteria, fungi, protozoa, mycoplasma, and viruses (Mardha KT et al., 1998). The majority of infections in the female reproductive tract (FRT) occur in the vagina and cervix. Numerous microorganisms are often linked to cervical infection, leading to cervicitis, including herpes simplex virus-2 (HSV-2), *Chlamydia trachomatis* (CT), *Neisseria gonorrheae* (NG), and Mycoplasma (Laniewski et al., 2020). The most common forms of vaginitis include desquamative inflammatory vaginitis (DIV) or aerobic vaginitis (AV), bacterial vaginosis (BV), vulvovaginal candidiasis (VVC), cytolytic vaginosis (CV) and trichomonas vaginalis (TV). Mixed vaginitis in this review encompasses these common types of vaginitis. The signs and symptoms of mixed vaginitis are often atypical, treatment is complicated in contrast to single-type vaginitis. It is largely ignored and poorly studied. Therefore, the major goal of this review is to help improve clinicians’ understanding of mixed vaginitis and discuss the therapeutic standard to reduce the disease burden and prevent associated complications.

## Method

Search Methods. A systematic search was performed in the PubMed database; search results were not limited by publication year. Search strings included the following: “mixed vaginitis”, “mixed vaginal infection”, “mixed biofilms”, “polymicrobial infections”, “vulvovaginal candidiasis”, “bacterial vaginosis”, “trichomoniasis”, “aerobic vaginitis”, “desquamative inflammatory vaginitis” and “cytolytic vaginosis”. Additional potentially relevant studies that were not identified by the database search were identified by examining the references of the selected clinical studies and review articles. A total of 75 studies on mixed vaginitis were identified in our literature search.

Selection Criteria. The following inclusion criteria were used for study selection: studies on any type of vaginitis with information about clinical features, diagnosis and treatment; studies that reported the incidence of mixed vaginitis but we only choose the epidemiologic data of mixed vaginitis in the last 10 years; and studies on polymicrobial interactions. The exclusion criteria were as follows: studies not in English, letters, case reports or conference abstracts; only have the epidemiologic data of singe vaginitis were excluded in the epidemiologic data.

## Epidemiology

Although vaginitis is common, affecting millions of women every year, little information about the prevalence of mixed vaginitis is available. A literature review to assess the occurrence and frequency of mixed vaginitis revealed that the proportion of mixed vaginitis ranged from 4.44% to 35.06% (Rivers et al., 2011; Wang et al., 2017). The representative data are depicted in **Table 1**. The following factors are limitations that prevent the obtention of a clear picture of the actual prevalence of mixed vaginitis.

**Table 1:** Summary of representative data of mixed vaginitis in the last 10 years.

Year	Author	Area	Patients with vaginitis	Rate of mixed vaginitis	AV+BV	VVC+BV	TV+BV	VVC+AV	AV+TV	VVC+TV	Multiple vaginitis	IF	Diagnosticcriteria
2021 (Vieira-Baptista et al., 2021)	Vieira-Baptista P	Portugal	277	21 (7.58%)		16 (76.19%)	5 (23.81)					6.53	NAATs
2021 (Hillier et al., 2021)	Hillier SL	US	170	29 (17.06%)		17 (58.62%)	10 (34.48%)			1 (3.45%)	1 (3.45%)	8.31	RM and NAATs
2020 (Pacha-Herrera et al., 2020)	Pacha-Herrera D	Ecuador	89	4 (4.49%)	2 (50%)	1 (25%)		1 (25%)				5.29	RM
2020 (Schwebke et al., 2020)	Schwebke JR	US	940	256 (27.23%)		147 (57.42%)	71 (27.73%)			15 (5.86%)	23 (15.65%)	4.96	NAATs
2020 (Salinas et al., 2020)	Salinas AM	Ecuadorian	95	16 (16.84)								3.99	RM
2020 (Donders et al., 2020)	Donders GG	Belgium	250 (RVVC)	97 (38.8%)		22 (22.68%)		75 (77.32%)				2.49	RM
2020 (Elkins et al., 2020)	Elkins JM	US	5802	316 (5.45%)		136 (43.04%)	150 (47.46%)			20 (6.33%)	10 (3.16%)	1.2	RM
2019 (Kamga et al., 2019)	Kamga YM	Cameroon	198	28(14.14%)		28(100.00%)	0 (0.00%)					2.41	RM
2019 (Sherrard, 2019)	Sherrard J.	UK	186	15(8.07%)		14 (93.30%)				1 (6.70%)		1.50	RM
2019 (Sherrard, 2019)	Sherrard J.	UK	172	36 (20.93%)		36 (100.00%)						1.50	NAATs
2019 (Khan et al., 2019)	Khan Z	India	247	21 (8.50%)		21 (100.00%)	0 (0.00%)			0 (0.00%)		2.38	RM
2019 (Abdul-Aziz et al., 2019)	Abdul-Aziz M	Yemen	130	10 (7.69%)		9 (90.00%)				1 (10.00%)		2.56	RM
2019 (Konadu et al., 2019)	Konadu DG	Ghana	332	74 (22.29%)		67(90.54%)	3(4.05%)			4 (5.41%)		2.41	RM
2017 (Gaydos et al., 2017)	Gaydos CA	America	1118	289 (25.85%)		195 (67.47%)	64 (22.15%)			7 (2.42%)	23 (7.96%)	4.97	RM
2017 (Venugopal et al., 2017)	Venugopal S	India	77	3 (3.89%)		3 (100.00%)						1.36	RM
2017 (Carrillo-Avila et al., 2017)	Carrillo-Avila JA	Granada	29 (TV)	12 (41.38%)			7 (58.33%)			3 (25%)	2 (16.67%)	2.16	NAATs
2017 (Wang et al., 2017)	Wand HX	Shanghai	4036	1415 (35.06%)		606 (42.83%)	471(33.27%)					1.50	RM
2016 (Liang et al., 2016)	Liang Q	Tianjin	142 (AV)	84 (59.15%)	36 (42.85%)			26 (30.95%)	22 (26.19%)			1.39	RM
2016 (Byun et al., 2016)	Byun SW	Korea	108	10 (9.26%)								1.12	NAATs
2016 (Wang et al., 2016)	Wang ZL	Chongqing	830	184 (22.17%)	101 (54.90%)			48 (26.10%)	15 (8.20%)		20 (10.80%)	1.20	RM
2013 (Jahic et al., 2013)	Jahic M	Sapna	96	30 (31.30%)	8 (26.70%)			13 (43.30%)	9 (30.00%)			0.00	RM
2013 (Fan et al., 2013)	Fan A	Tianjin	657	170 (25.88%)	31 (18.24%)	62 (36.47%)	18 (10.59%)	32(18.82%)	21(12.35%)	1 (0.58%)	5 (2.94%)	2.28	RM
2012 (Bohbot et al., 2012)	Bohbot JM	France	118	38 (32.20%)								0.00	Unspecified
2011 (Rivers et al., 2011)	Rivers CA	Birmingham	338	15 (4.44%)		15(100.00%)						2.27	RM
2011 (Gondo et al., 2011)	Gondo F	Brazil	112	7 (6.30%)		7 (100.00%)						1.30	RM

①The “patients with vaginitis “ in column 4 refers to the women having a laboratory-diagnosed cause of vaginitis; “250 (RVVC)” refers to 250 patients with RVVC; “29 (TV)” refers to 29 patients with TV; “142 (AV)” refers to 142 patients with AV; ②The “rate of mixed vaginitis” in column 5 refers to the ratio of mixed vaginitis to total vaginitis; ③The rates in the following columns refer to the ratio of each item in mixed vaginitis; ④The “Diagnostic criteria” in column 14 refers to RM and NAAT. RM: The reference methods for BV were Nugent’s score and Amsel’s criteria. The reference methods for VVC and TV were wet mount and culture. The reference method for AV was wet mount based on the criterion by Donders. NAAT, Nucleic acid amplification.

The types of vaginitis observed have not been concordant. Evaluations have traditionally focused on VVC, BV, and TV. Most studies have reported that VVC plus BV is the most prevalent form of vaginitis (Rivers et al., 2011). In addition, it is possible that some clinicians are unaware of DIV/AV, thus sometimes misdiagnosing it as BV, affecting the epidemiological data. When DIV/AV is included, epidemiologic estimates shift considerably. Some studies indicated that DIV/AV plus BV, VVC plus DIV/AV, and VVC plus BV were the most frequent forms of mixed vaginitis (Fan et al., 2013).

There is great variability in the rates of mixed vaginitis in different populations. One study found a relatively low rate of mixed vaginitis (4.44%) in Brazil (Rivers et al., 2011), while another found a higher rate (35.06%) in Shanghai (Wang et al., 2017). Research is required to demonstrate prevalence and outcomes in various populations, such as pregnant women, hypoestrogenic women, asymptomatic women, and so on.

The diagnostic criteria and tools to determine the prevalence of mixed vaginitis differ. The classical standards for vaginitis diagnosis are physical examination, microscopy, and culture methods, which are usually performed in hospitals. The skill level of technicians is an influencing factor (Nyirjesy et al., 2020). Recent research has shown that some new molecular assays (Affirm VPIII, Aptima, BD Max, Seegene Allplex) for the diagnosis of mixed vaginitis have performed well, identifying proportions ranging from 7.58% to 27.23% (Schwebke et al., 2020; Vieira-Baptista et al., 2021).

The vaginal and cervical microbiome is an intricate ecosystem containing various microbes in different ratios. At present, in the mixed vaginitis-related literature, only 5 common types of vaginitis are included. If one includes the cervical, but not strictly vaginal, pathogens such as HSV-2 virus, CT, NG, mycoplasma, and HPV may be included, and higher frequencies of mixed infections may be reported (Brotman et al., 2010).

There is a lack of physician understanding and implementation of current guidelines (Nyirjesy et al., 2020). This is likely due, in part, to the fact that the majority of these infections are diagnosed empirically without objective data. Moreover, mixed vaginitis symptoms can be nonspecific and vary by patient. Empirical evidence in this population has likely led to many misdiagnoses.

## Mechanism of Mixed Vaginitis

Multiple microorganisms generally involve the formation of mixed biofilms, dominated by bacteria and/or fungi, embedded in an extracellular matrix (Beaussart et al., 2013). The specific characteristics of mixed biofilms, especially their enhanced drug resistance and their ability to evade components of the host immune response, make them of high clinical importance. However, despite the importance of such mixed biofilms, mixed biofilms research, particularly research involving vastly interspecies interactions, is in its infancy (Schlecht et al., 2015). Bacteria and/or fungi can coexist within a host, and the nature of interspecies interactions can determine the fate of microbial populations. They influence each other in diverse ways *via* synergistic or antagonistic interactions (Lohse et al., 2018).

Medically antagonistic interactions between microorganism are common in the lower female reproductive tract. It is more likely to occur between probiotics and pathogens. For example, studies on the vaginal microbiota have revealed that *Lactobacillus* species lower the local pH (by releasing lactic acid), which results in the inhibition of initial adherence of *Candida albicans* and *Gardnerella vaginalis* to the vaginal mucosal surface (Deidda et al., 2016). Many environmental cues impact biofilm formation, such as hypoxia, elevated extracellular pH, body temperature, and elevated CO2 (Castro et al., 2016). Similarly, a previous study reported that *Lactobacillus* in healthy women vaginally disrupted *Gardnerella* biofilm surface area, density and depth (Saunders et al., 2007). Little is known about pathophysiological vaginal conditions during mixed vaginitis, so the structure and composition of the mixed biofilm to understand mixed vaginitis needs to be further explored.

Some synergistic relationships result in complex pathogenic processes, providing protection to one or both species in mixed-species biofilms. It is more likely to occur between pathogens. This occurs in the following ways: cells of certain species can directly bind to cells of other species. For example, recent evidence has indicated that *Staphylococcus aureus* can “piggyback” on *C. albicans* hyphae to penetrate host cells, infiltrate deep tissues and participate in the pathogenic process of host cells (Schlecht et al., 2015). Similar synergies providing a protective microenvironment have also been observed; for example, the presence of a *C. albicans* biofilm enables the proliferation of anaerobic pathogens in an otherwise hostile, oxygen-rich environment. Moreover, the bacteria seem to induce the formation of these protective structures (Fox et al., 2014). A recent study linked this protective interaction to enhanced drug resistance; when *C. albicans* and methicillin-resistant *Staphylococcus aureus* (MRSA) strains were grown together, the presence of *C. albicans* seemed to protect MRSA from eradication by vancomycin (Harriott and Noverr, 2010). Synergistic interactions can also enhance virulence during infection (Morales et al., 2013). For example, higher host mortality was observed when *S. aureus* and *C. albicans* were introduced together at sublethal doses in a mouse peritonitis infection model than when either species was introduced alone (Peters and Noverr, 2013). A limitation of these studies was that this interaction was evaluated not in the lower female reproductive tract. However, these observations illustrate the dynamic nature of polymicrobial interactions in part. In other words, the contemporaneous process may be interdependent. The mechanisms behind these synergistic interactions have not been described.

**Figure 1:**
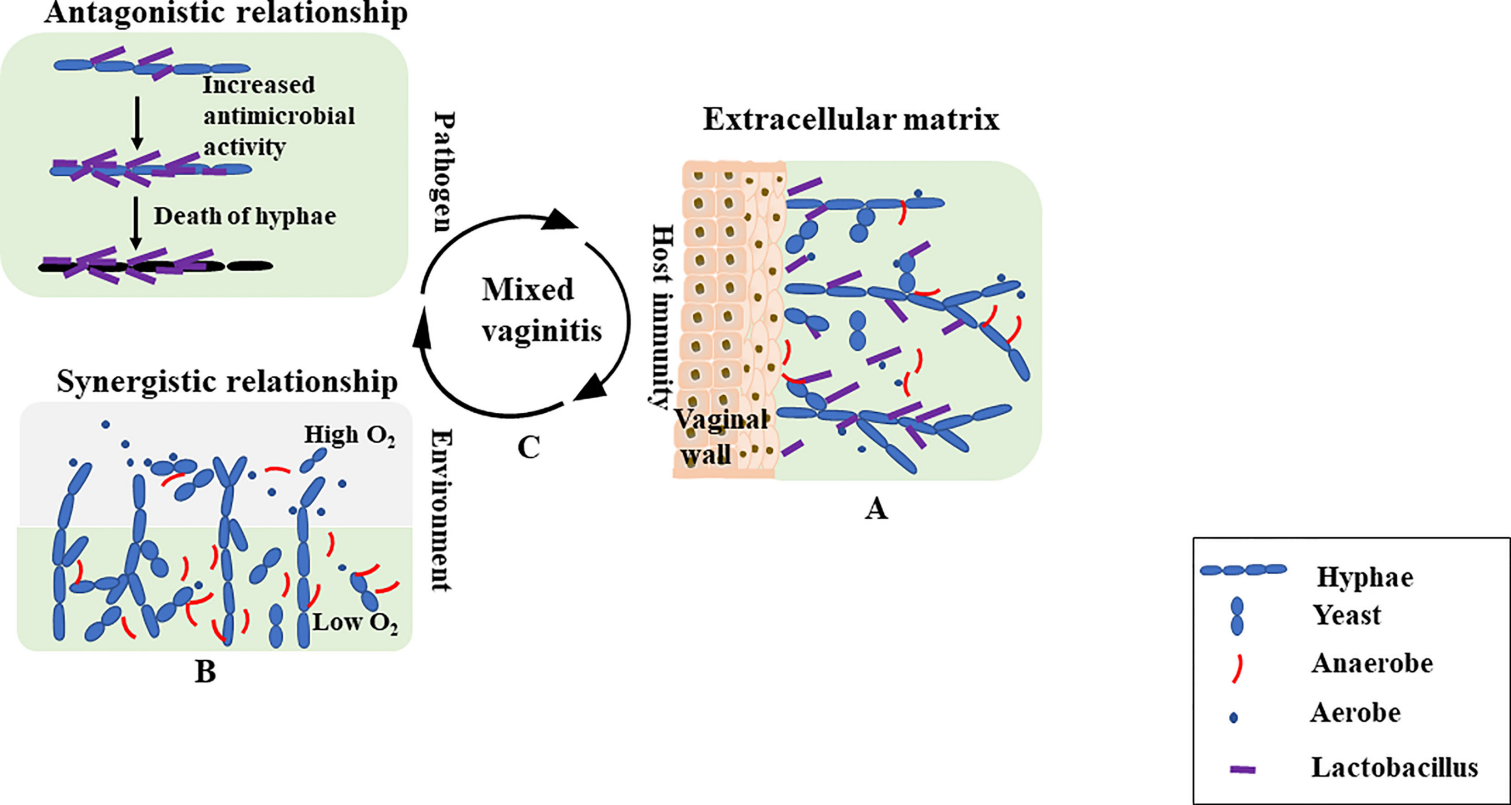
**(A)** The mixed-biofilms are complex structures in which bacteria and/or fungi adhere to the vaginal wall surfaces, and they are encased in an extracellular matrix. The extracellular matrix is a physical barrier to the outside environment. **(B)** Microbes within the biofilm also exhibit antagonistic and synergistic interactions. *Lactobacilli* can attach to the surface of *Candida albicans hyphae*. Production of antimicrobials leads to the death of fungal filaments. In addition to antagonistic interactions, mutually beneficial interactions in mixed biofilm environments are also possible. For example, *C. albicans* can protect anaerobic bacteria by providing a low oxygen niche within the depths of the biofilm, even though the external environment is aerobic. **(C)** Antagonistic interactions are more likely to occur between probiotics and pathogens. Synergistic interactions are more likely to occur between pathogens. Different microbes can determine the course of mixed vaginitis. In interspecies interactions, many environmental cues (hypoxia, extracellular pH, body temperature and CO_2_) and host immune factors could impact the formation of mixed vaginitis.

We have focused on antagonistic versus synergistic interactions, but additional distinct interactions exist. Multiple microorganisms challenge the immune system in different ways compared with single microbe. A host response to one microbe may promote the proliferation of another microbe. For example, coinfection with *Streptococcus agalactiae* significantly attenuated the hyphal development of *C. albicans in vitro*, but it may attenuate host vaginal mucosal TH17 immunity and contribute to mucosal colonization by *C. albicans in vivo* (Yu et al., 2018). A multicountry cross-sectional study reported that the factor independently associated with *S. agalactiae* was *C. albicans* presence (Cools et al., 2016). Similarly, another study suggested that *C. albicans* may suppress the local host immune response, allowing subclinical *P. aeruginosa* to proliferate, resulting in disease (Roux et al., 2009) (**Figure 1**). Thus, these interactions are highly complex, and the type of interaction that occurs often depends on a range of environmental, pathogenic and host factors. The mechanisms of mixed vaginitis are unknown thus far, and further exploration is needed.

## Clinical Features

In single vaginitis, different types of vaginitis have different vaginal milieu and clinical manifestations. It is essential to compare the clinical manifestations and microorganisms in various vaginitis in order to recognize the mixed vaginitis. The representative content is depicted in **Table 2**. Mixed vaginitis may be atypical. It can be characterized by single vaginitis and can also be characterized by the simultaneous presence of two or more potential vaginitis features (Mardha KT et al., 1998; Fan et al., 2013). For example, patients with AV plus BV reported a genital fish-like odor more than a single AV and an inflammation more than a single BV. Patients with AV plus VVC more often reported genital itching than those with a single AV (Fan et al., 2013). Symptoms varied among the patients with mixed vaginitis. The most frequently reported symptoms included a change in the characteristics of discharge (color, consistency, odor), genital itching, and burning pain. Additionally, mixed vaginitis may be hard to eradicate, and recurrence is frequent. For example, fenticonazole was evaluated in a study, the eradication rate of mixed vaginitis was lower, and the relapse rate was higher than that of single vaginitis (Fabio Tumietto, 2019). This is likely due to the diverse behavior of the pathogenic vaginal flora that seems to affect the immune response of the host, making cure difficult.

**Table 2:** A comparing the clinical manifestations and opportunistic pathogens in vaginitis.

		microbe	Pathogenic factor	Clinical manifestations
AV/DIV	Endogenous infection	Streptococcus spp., S. aureus, Group B streptococci, E. coli and E. faecalis	Host immune status More sex partners Hygiene practices Intrauterine device (IUD) Antibiotic therapy Immunosuppression	Approximately10–20% asymptomatic. Symptoms: inflammation, introital and vaginal redness, stinging and burning sensations, the presence of sticky, yellow vaginal discharge and dyspareunia. Signs: vaginal discharge is described as homogeneous and purulent, yellowish or yellow-green in color.
BV		G. vaginalis, Prevotella spp., A. vaginae, Megasphaera type 1, and numerous other fastidious or uncultivated anaerobes		Approx. 50% asymptomatic. Symptoms: a fishy odor of vaginal discharge. Signs: homogeneous, thin discharge (milk-like consistency) that smoothly coats the vaginal walls.
VVC		Candida albicans Non–albicans Candidiasis		Approx. 60% women colonized. Minority develop symptoms. Symptoms: external dysuria and vulvar pruritus, pain, swelling, and redness. Signs: vulvar edema, fissures, excoriations, and thick curdy vaginal discharge.
CV		Lactobacillus spp.	Estrogen	Symptoms and Signs are overlap with VVC.
TV	Exogenous infection	Trichomonas vaginalis	Sexual transmission Public baths and articles	Approx.10–50% asymptomatic and 5–15% no abnormal signs. Symptoms: vaginal discharge which can be diffuse, malodorous, or yellow-green with or without vulvar irritation. Signs: inflammation, introital and vaginal redness, stinging and burning sensations and a strawberry-appearing cervix.
MV	Endogenous add/or exogenous infection	Simultaneous presence of at least two vaginal pathogens	Host immune status Polymicrobial interactions	Symptoms and signs can be characterized by single vaginitis, can also simultaneous presence of two or more potential vaginitis features.

AV, aerobic vaginitis; DIV, severe AV is desquamative inflammatory vaginitis; BV, bacterial vaginosis; VVC, vulvovaginal candidiasis; CV, cytolytic vaginosis; TV, trichomoniasis; MV, mixed vaginitis.

## Diagnosis of Mixed Vaginitis

**Figure 2:**
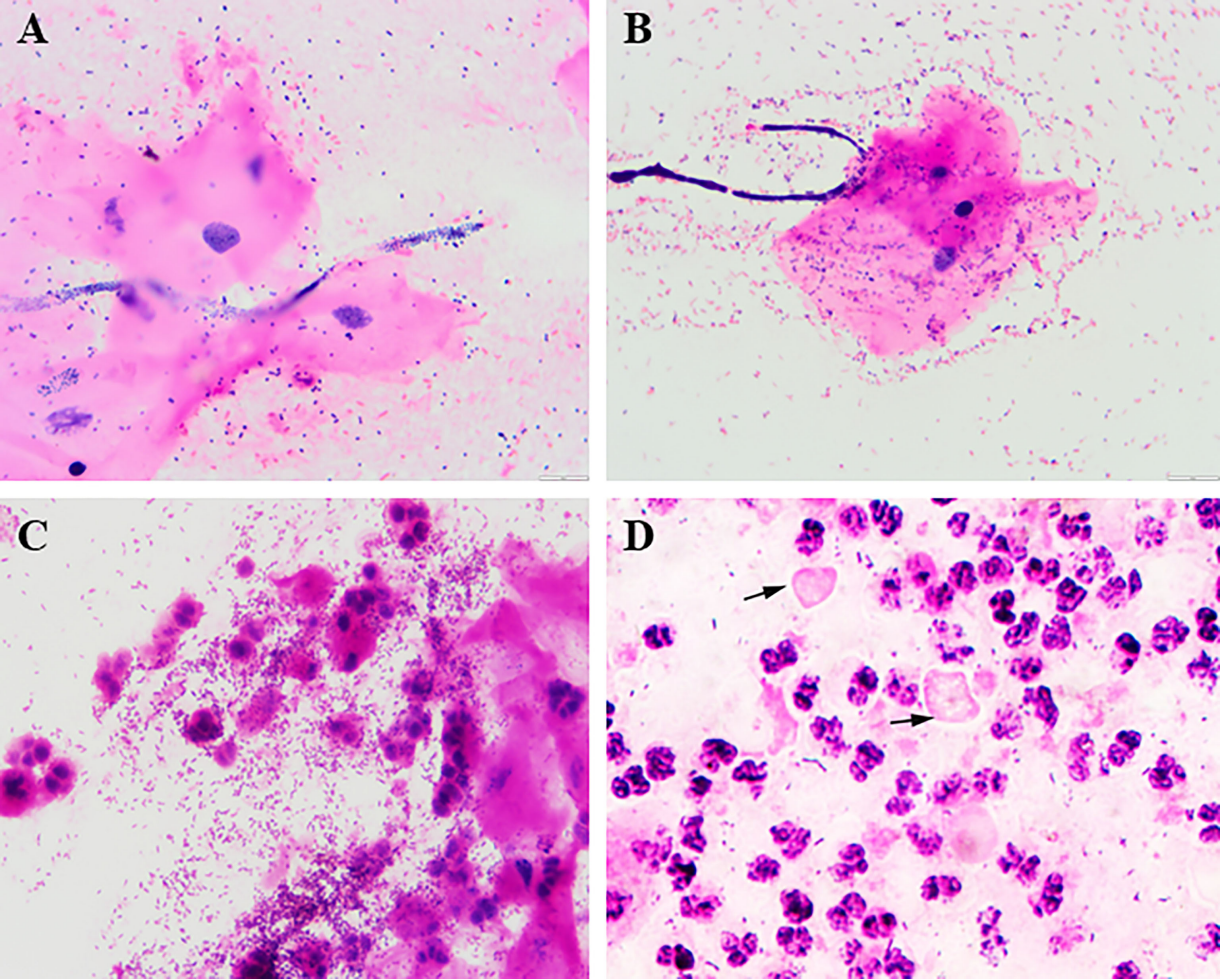
Mixed vaginitis under Gram staining smears (1000×). **(A)** *Candida* + *coccus*; **(B)** *Candida* + BV; **(C)** BV + inflammation; **(D)** TV + DIV/AV; arrows indicate *Trichomonas*.

A mixed vaginitis diagnosis is made according to the presence of symptoms, clinical findings and laboratory tests (Gram-staining, wet-mount smears, PCR tests and combination of point-of-care tests) (Tempera et al., 2004). Some studies confirm that the presence of multiple vaginitis did not interfere with the assay performance (Vieira-Baptista et al., 2021). The key points in diagnosing mixed vaginitis are as follows (Sobel et al., 2013): an abnormal vaginal milieu and the simultaneous presence of at least two types of vaginitis. Since the diagnosis of mixed vaginitis is largely dependent on the diagnostic criteria for single vaginitis, the criteria to facilitate recognition of the coexistence of multiple pathogens are as follows. Microphotographs of Gram-staining smear with mixed vaginitis are shown in **Figure 2**.

**DIV/AV:** The diagnosis of DIV/AV should be based on a combination of clinical features and microscopic findings (Oerlemans et al., 2020). The clinical features are as follows: vulvar erythema; vulvar swelling; thinning of the vaginal mucosa; vaginal congestion; scattered bleeding points; and yellow-colored vaginal secretion, increased discharge or pruritus. The microscopic features were as follows: wet mount smears with an DIV/AV score ≥3 (Tempera et al., 2004). Accordingly, three main characteristics according to the guidelines from “Vaginitis and Microbiome Committee” of the International Society (Vieira-Baptista et al., 2021): ratio leukocytes: epithelial cells of greater than 1; presence of parabasal cells; and a disturbed bacterial community lacking the commonly observed high abundance of lactobacilli. Culture is not recommended for diagnosis, and a positive vaginal culture does not indicate the woman has DIV/AV. However, culture may be useful for treatment (Sherrard et al., 2018). In addition, some groups have begun to develop a nucleic acid amplification test (NAAT) to circumvent microscopic defects. However, the detailed information obtained with phase contrast microscopy is irreplaceable because it is still unclear whether DIV/AV is an “infection” or “dysbiosis”.

**BV:** at least one of the following must be present: a Nugent score (Nugent et al., 1991) >6; the Nugent score is considered the gold standard for studies and relies upon estimating the relative proportions of bacterial morphotypes on a gram-stained vaginal smear to assign a score between 0 and 10. The presence of three of four Amsel’s criteria, including homogeneous, thin, white discharge that smoothly coats the vaginal wall; clue-cells on microscopic examination (prerequisite); pH of vaginal fluid >4.5; or vaginal discharge with a fishy odor before or after the addition of 10% KOH (whiff test). Amsel’s criteria have a sensitivity of 37–70% compared to the Nugent score, and has a poor performance for the diagnosis of BV (Vieira-Baptista et al., 2021). Moreover, multiple point-of-care (POC) tests are available for BV diagnosis. The Osom BV Blue test (Sekisui Diagnostics, Framingham, MA, USA) detects vaginal sialidase activity. This test has been reported to be most useful for symptomatic women in conjunction with vaginal pH and amine odor. In addition to the POC test, multiple BV NAATs are available among symptomatic women. Commercial tests (BD Max Vaginal Panel, Aptima BV and Seegene Allplex, etc.) are performed very well. NAATs will be the future for the diagnosis of BV but traditional methods of BV diagnosis, including the POC tests and Nugent score, remain useful because of their lower cost and rapid diagnosis (Workowski et al., 2021).

**VVC:** at least one of the following must be present: the presence of yeast or pseudohyphae in vaginal discharge on wet-mount microscopy with either saline or 10–20% KOH solution (40–60% sensitivity); the presence of yeasts or pseudohyphae on gram staining (up to 65% sensitivity) of vaginal discharge; or positivity on culture, which is helpful in diagnosing recurrent or complicated vulvovaginal candidiasis because species other than *C. albicans* (e.g., *Candida glabrata*, *Candida tropicalis*) may be present (Sherrard et al., 2018).

**CV**: The diagnosis of CV should be based on a combination of clinical features and microscopic findings. The signs and symptoms of CV are as follows (Hacısalihoğlu and Acet, 2021): vulvar and vaginal itching and burning, entry dyspareunia and a scant amount of white, frothy or cheesy vaginal discharge. The laboratory diagnosis follows a Cibley’s criteria: absence of *Trichomonas*, *Gardnerella* or *Candida*; an increased number of lactobacilli (often adherent to the intermediate epithelial cell); a paucity of white cells; evidence of cytolysis with bare or naked intermediate nuclei; and a pH between 3.5 to 4.5 (Cibley and Cibley, 1991).

**TV**: at least one of the following must be present: positivity on wet-mount smear, although the sensitivity has been reported to be as low as 45–60% (Nye et al., 2009); positivity on culture, which has a higher sensitivity than microscopy but is not widely available in clinical settings; or positivity on NAAT, which has the highest sensitivity for the detection of TV in comparison to both microscopy and culture. The Guidelines Group recommends that the most effective tests to diagnose TV in women are NAATs (Sherrard et al., 2018). However, examination of wet-mount preparations is still commonly used in clinical practice.

## Limitations of Mixed Vaginitis Diagnosis

Although clinical laboratory testing and clinical findings can identify the mixed vaginitis cause in the majority of women, inaccurate diagnosis or failure to recognize mixed vaginitis may occur in the following situations. Given the overlap between the signs and symptoms in various vaginitis, it is difficult to draw firm conclusions. For example, a yellow or green-yellow discharge was usually observed in patients with a single DIV/AV. However, patients with DIV/AV mixed vaginitis (e.g., DIV/AV plus BV, DIV/AV plus VVC) usually also report a green-yellow, thin, purulent vaginal discharge. Another reason for misdiagnosis is coinfection with cervical pathogens. Cervicitis frequently is asymptomatic; however, certain women might report an abnormal vaginal discharge. When a patient has inflammation along with BV, we must suspect that cervicitis/PID or TV is also present, and women with TV sometimes might have a strawberry-appearing cervix. Therefore, amalgamative infection of the cervical and vagina should be recognized. Some cervical infections caused by pathogens, such as HSV-2, CT, NG, and mycoplasma (Curry et al., 2019), might occur concurrently with vaginitis, and symptoms of cervicitis are generally obscured, increasing the complexity of diagnosis. Thus, coinfection with the pathogens mentioned above should be excluded in the diagnosis of mixed vaginitis. Diagnostic testing for cervical infections should be performed for persons with mucous purulent discharge.

## Treatment

Mixed vaginitis poses a therapeutic challenge. Consideration for polytherapy is appropriate for consecutive symptoms and signs in mixed vaginitis (e.g., symptomatic BV in patients following treatment of VVC; symptomatic VVC follows treatment of TV). However, polytherapy is unnecessary, particularly involving asymptomatic colonized microbes. Therefore, many countries have banned the availability of combination antimicrobial products for use in vaginitis. Standard treatment for mixed vaginitis has not yet been established. Although current in-clinic methods for the treatment of mixed vaginitis present wide variation, the treatment principles state the following: 1) according to microorganisms and pathogenesis, (e.g., treatment of DIV/AV adapted to the presence of three different components: infection, inflammation or atrophy) standard treatments should be chosen to minimize the abuse of unnecessary antibacterial drugs (Donders et al., 2017); 2) we should treat sexually transmitted infection first when the simultaneous presence of sexually transmitted disease (such as TV), meanwhile treating their sexual partners (Workowski and Bolan, 2015); and 3) we should treat single vaginitis with severe symptoms first when there is a contradiction in medication (Han et al., 2015) (such as treatment of DIV/AV plus VVC, if started by treating VVC, usually the symptoms disappear, and the vaginal flora changes to a more “normal” state). 4) Lay emphasis on alleviating patients’ symptoms as soon as possible. One challenge is that individual signs and symptoms have shown only modest value in diagnosing mixed vaginitis. Therefore, how to identify at-polytherapy subpopulations requires further consideration.

Although anti-infective treatments are available and are usually highly efficient in eradicating microorganisms, the long-term efficiency is hampered by relapse (Cohen et al., 2020). How to reduce the recurrence of mixed vaginitis needs further exploration and confirmation in larger trials. The main treatment objectives are the alleviation of symptoms, the elimination of pathogens, and eventually the recovery from disturbed to healthy lactobacilli-dominated vaginal flora.

This review summarized the representative epidemiological data of mixed vaginitis and suggested that mixed vaginitis is a common cause of vulvovaginal symptoms. In contrast to research on single vaginitis, research on mixed vaginitis is still in the preliminary stage. Mixed vaginitis remains understudied and underrecognized. The pathogenic mechanism of mixed vaginitis needs to be further explored. Mixed vaginitis generally involves the formation of mixed biofilms. The study of polymicrobial interactions and mixed biofilms will provide a new idea for the understanding of mixed vaginitis. The nature of interspecies interactions can determine the fate of microbial populations. Thus, it appears possible to utilize these interactions for prophylactic and therapeutic within the host. Moreover, effective management of mixed vaginitis depends on laboratory diagnosis to avoid inappropriate therapy, recurrence, and reinfection. Although two types of vaginitis may be identified, a potential vaginitis may be present but may not be the cause of existing vaginal symptoms. Therefore, the accuracy of diagnosis and how to identify at-polytherapy subpopulations requires further consideration. In summary, this review is of great importance for improving clinical awareness of mixed vaginitis and facilitating female reproductive health.

## Author Contributions

The author is grateful to Xue F and Han C, who provided suggestions for this review. Li H, Wang C, and Fan A made substantial contributions to drafting the article and revising it critically. Furthermore, all authors have given their final approval for this version to be published and agree to be accountable for all aspects of the work.

## Funding

This work was supported by National Natural Science Foundation of China (No. 82101705), Tianjin Health Science and Technology Project (Grant number QN20034, KJ20176 and KJ20003) and Scientific Research Project of Tianjin Education Commission (Grant number 2020KJ158).

## Conflict of Interest

The authors declare that the research was conducted in the absence of any commercial or financial relationships that could be construed as a potential conflict of interest.

## Publisher’s Note

All claims expressed in this article are solely those of the authors and do not necessarily represent those of their affiliated organizations, or those of the publisher, the editors and the reviewers. Any product that may be evaluated in this article, or claim that may be made by its manufacturer, is not guaranteed or endorsed by the publisher.
